# Prospective on Imaging Mass Spectrometry in Clinical Diagnostics

**DOI:** 10.1016/j.mcpro.2023.100576

**Published:** 2023-05-19

**Authors:** Jessica L. Moore, Nathan Heath Patterson, Jeremy L. Norris, Richard M. Caprioli

**Affiliations:** 1 Frontier Diagnostics, Nashville, Tennessee, USA; 2 Vanderbilt University Mass Spectrometry Research Center, Vanderbilt University, Nashville, Tennessee, USA; 3 Departments of Biochemistry, Pharmacology, Chemistry, and Medicine, Vanderbilt University, Nashville, Tennessee, USA

**Keywords:** imaging, imaging mass spectrometry, MALDI MS, clinical diagnostics, clinical proteomics

## Abstract

Imaging mass spectrometry (IMS) is a molecular technology utilized for spatially driven research, providing molecular maps from tissue sections. This article reviews matrix-assisted laser desorption ionization (MALDI) IMS and its progress as a primary tool in the clinical laboratory. MALDI mass spectrometry has been used to classify bacteria and perform other bulk analyses for plate-based assays for many years. However, the clinical application of spatial data within a tissue biopsy for diagnoses and prognoses is still an emerging opportunity in molecular diagnostics. This work considers spatially driven mass spectrometry approaches for clinical diagnostics and addresses aspects of new imaging-based assays that include analyte selection, quality control/assurance metrics, data reproducibility, data classification, and data scoring. It is necessary to implement these tasks for the rigorous translation of IMS to the clinical laboratory; however, this requires detailed standardized protocols for introducing IMS into the clinical laboratory to deliver reliable and reproducible results that inform and guide patient care.

## Highlights

• We review the use of IMS for the development of new molecular assays
• We discuss quality control and quality assurance metrics for IMS assays
• We explore IMS for diagnostic and prognostic clinical applications
• We discuss regulatory requirements necessary for practical implementation

## Graphical Abstract

**Figure:**
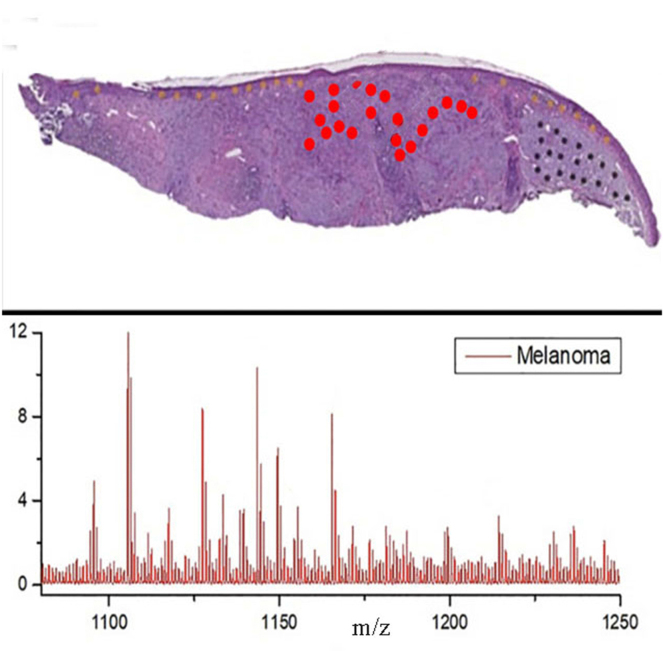

## In Brief

Imaging mass spectrometry is an emerging technology for clinical applications in that it provides spatial molecular data within a tissue biopsy for patient diagnoses and prognoses. This article considers various aspects of the use of this technology for new imaging-based assays that include analyte selection, quality control/assurance metrics, data reproducibility, data classification, and data scoring.

Society is well into a molecular age in medicine, one promising the capability to understand disease at the molecular level and either eliminate or mitigate the effects. However, the molecular expression from genomic, proteomic, and metabolomic processes ongoing in living cells is enormously complex, continuously challenging our ability to measure and understand their integrated totality. Seminal advances in clinical effectiveness in diagnosing and treating disease have often been preceded by innovations in the technology used to probe cells and tissue biopsies. Disease diagnoses, prognoses, and the assessment of the therapeutic interventions will be most effectively done using multiplex molecular markers made up of protein, lipid, or small metabolites that constitute a molecular signature of the disease or related disease outcome (1, 2, 3). Single biomarkers are poor predictors because of the myriad biological interactions in cells.

Nowhere is this molecular description of disease needed more than in the histology/pathology laboratory, where the assessments of biopsies have life-changing implications. Over the years, the experience and diligence of pathologists has provided a body of knowledge using microscopy and various staining protocols. Immunohistochemistry has added a desperately needed molecular dimension to modern pathology, although its shortcomings are well known and troublesome. Antibodies are only surrogate markers of molecules of interest, and their effectiveness depends on their specificity for a given antigen. Multiple studies have shown that many antibodies recognize more than one antigen and their specificity for other molecules is often unknown (4). Furthermore, evaluation of the stained biopsy can vary widely because it is subjective, varying from observer to observer (5, 6). Direct analysis of many markers of disease is needed to give pathologists the molecular information they need to diagnose disease in today’s medical world.

**Fig. 1:**
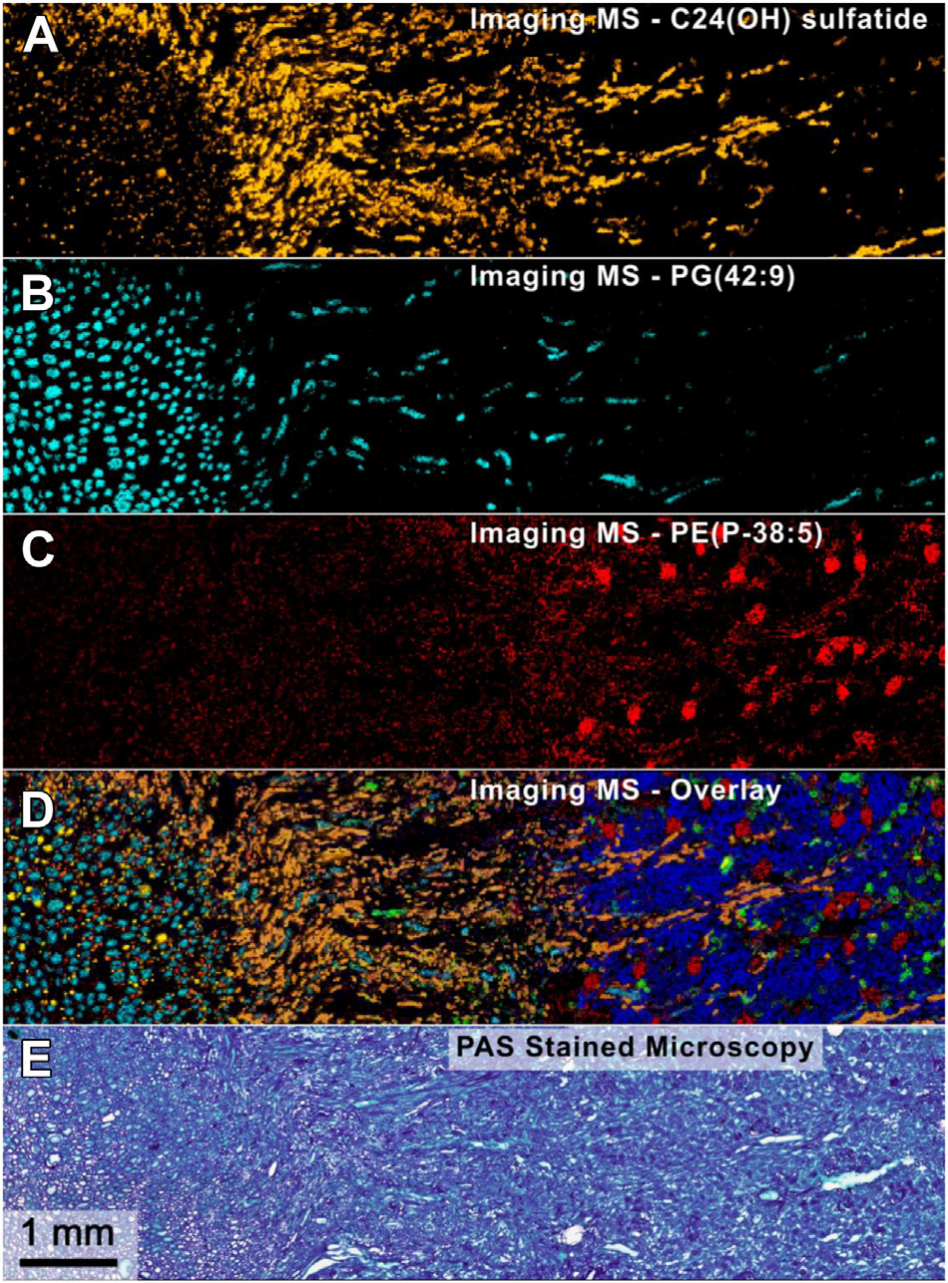
**MALDI imaging mass spectrometry (IMS) of human kidney tissue collected at 20-μm spatial resolution using 9.4T MALDI FT-ICR.***A*–*C*, spatial distributions of various lipids shown individually: C24(OH) Sulfatide (*orange*, *m/z* 906.634), PG(42:9) (cyan, *m/z* 843.518), PE(P-38:5) (*red*, *m/z* 748.529). *D*, overlaid ion images, including images from *A* to *C* and other lipids: PE(34:2) (*dark blue*, *m/z* 714.508), PI(P-36:1) (*green*, *m/z* 847.570), C24 Sulfatide (*yellow*, *m/z* 890.637). *E*, PAS-stained tissue microscopy image collected after IMS analysis. FT-ICR, Fourier transform Ion cyclotron resonance; PAS, periodic acid-schiff.

MALDI imaging mass spectrometry (IMS) is a maturing technology that brings unparalleled molecular specificity and sensitivity to create molecular image maps of molecules in tissues (7, 8). It can be employed in multiple modes: complete molecular imaging of an entire biopsy or a histology-directed manner where selected regions are analyzed. The complete image analysis of a biopsy produces a series of molecular maps, each presented at a molecular weight, measured as *m/z* values (Fig. 1, *A*–*D*), from virtually any tissue sample. The technology uses laser ablation at spots or pixels on the target tissue uniformly arranged in an array across the field of interest (complete analysis) or discrete tissue areas (histology-directed analysis). The spatial resolution of the image is defined by the diameter of the ablation spot (typically 5–30 microns) and the pitch of the spots set by the user. Hundreds to thousands of discrete molecular maps can be generated from a single array acquisition. Microscopy images can be collected (Fig. 1*E*) and correlated with the new IMS-derived molecular maps. A pathologist can interpret these data with the assistance of unsupervised or supervised computer algorithms.

**Fig. 2:**
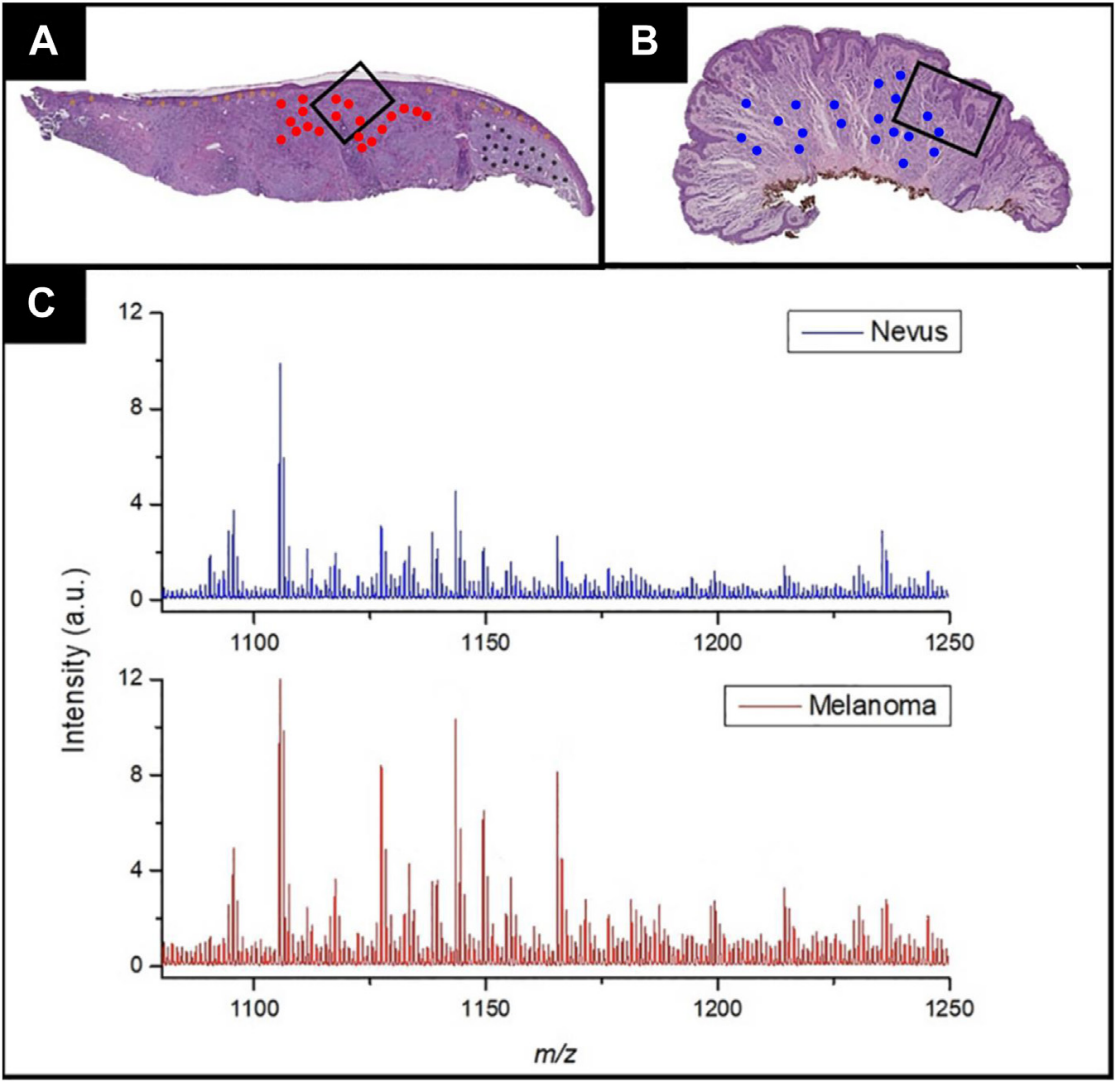
**Histology-directed MALDI imaging mass spectrometry (IMS) of melanocytic lesions in human skin biopsies embedded in FFPE for clinical diagnostics.** Individual spots were selected by a board-certified dermatopathologist, followed by *in situ* microdrop trypsinization of each spot to release peptides from proteins. *A*, low-magnification micrograph of an H&E stain of a human skin biopsy containing a melanocytic lesion with cancerous regions annotated by a red spot for histology-directed IMS analysis. *B*, low-magnification micrograph of an H&E stain of a human skin biopsy containing a benign lesion with benign growth regions annotated by a blue spot for histology-directed IMS analysis. *C*, peptide fingerprint mass spectra compare the red melanoma spots within the black box region in (*A*) and the benign blue spots within the black box in (*B*). This figure is modified from Reference (54). FFPE, formalin fixed paraffin embeded.

In clinical practice, pathologists may prefer the histology-directed mode, where discrete measurements are directed by the pathologist. In a typical application, validated disease cases and normal tissues are used in a model-building validation set of 50 to 100 biopsies to develop the molecular signatures of both diseased and normal tissue. Patient biopsies are then assessed for these signatures from each area marked by the pathologist. Figure 2 shows a histology-directed application for the diagnosis of malignant melanoma where IMS targeted melanocytic regions of tissue and a signature differentiating the two was developed. This example shows the difference in molecular expression measured when comparing normal and diseased tissue using IMS.

IMS provides pathologists with the tools to probe the molecular makeup of a biopsy directly without the limitations and expense of antibodies. Direct molecular mapping in disease is essential to provide greater molecular specificity and multiplexing. IMS represents a paradigm shift in the field of anatomic pathology that offers the pathologist new, precise molecular approaches to enhance the sensitivity of disease diagnosis.

**Fig. 3:**
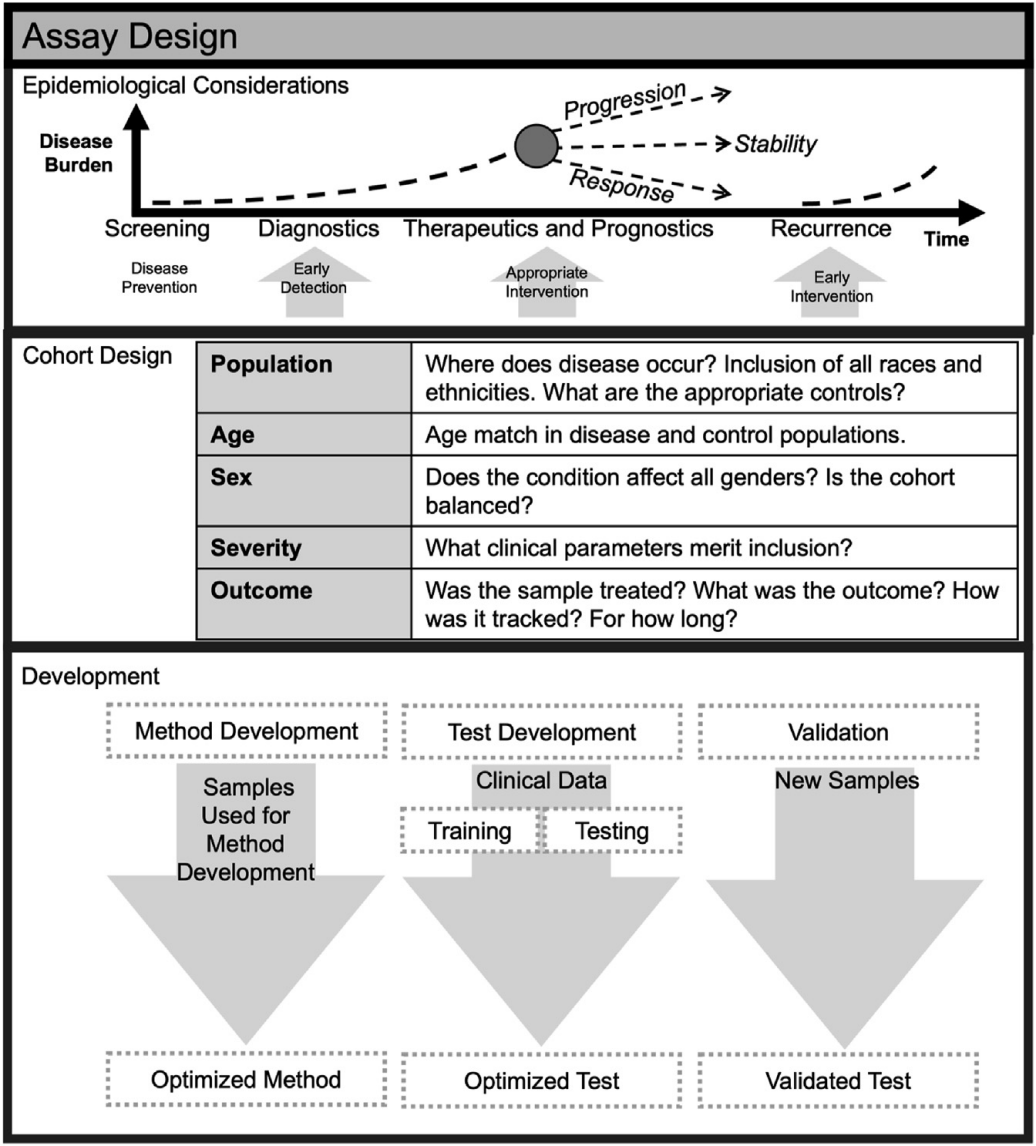
**High-level considerations when developing a clinical assay.** Overview of where a test fits into the clinical space, how to design an effective cohort, and the assay development cycle. *Top panel* adapted from Ludwig and Weinstein, *Nature Reviews*, 5, 2005.

This article considers IMS approaches for clinical diagnostics and essential factors for establishing successful clinical assays with this technology. These factors range from quality control in sample preparation to bioinformatics approaches. Aspects of new imaging-based assays include analyte selection, quality control/assurance metrics, data reproducibility, data classification, and data scoring. Areas of interest representing emergent challenges when using IMS for diagnostics and prognostics in clinical laboratories are considered. Figure 3 gives an overview of the epidemiological considerations, effective cohort design, and the general procedure for creating a validated test.

## Current MALDI mass spectrometry (Nonimaging) Applications in Clinical Laboratories

The precedent set by the US Food and Drug Administration approval of assays for identifying bacteria, *e.g.*, MALDI Biotyper (Bruker Corporation) and VITEK MS (bioMérieux SA), established widespread acceptance for the use of MALDI-TOF instrumentation in a clinical setting. MALDI-TOF systems are small, easy to use, and robust (9, 10). Whole-cell MALDI mass spectrometry (MS) from microbial colonies typically represents ribosomal proteins, providing a mass fingerprint for microbial identification (11, 12). Although the approach requires a microbial culture, this technology presents significant time and cost savings, ease of use, and unambiguous results (13). Overall, the performance of the MALDI assay exceeds that of the classical approaches to microbial ID in accuracy and speed.

Another emergent MALDI application for diagnostics is immuno-MALDI-TOF MS (iMALDI). These assays enrich samples using immunoaffinity before analysis by MALDI MS (14, 15). Enrichment can take place in solution using beads or antibodies immobilized in a pipette tip or directly on a surface (16). Enriched peptides can be eluted directly onto the MALDI target, minimizing sample loss due to handling tasks (15). Assays using iMALDI have detected glycosylated transferrin, a marker of alcoholism, from human serum (17) and screened human plasma for primary aldosteronism, which is clinically associated with hypertension (15, 18, 19). These assays have the potential to be multiplexed, further strengthening the applicability of these assays to clinical laboratories (15, 20, 21).

Plate-based MALDI assays have a throughput advantage. Nanoliter liquid handling systems can prepare targets that can analyze thousands of samples on a single target at a rate of 0.3 to 0.4 s per spot (22, 23, 24, 25). In a study by Vestal, 80 MALDI spots of the same blood sample were analyzed for glycosylated hemoglobin. The resulting standard deviation across the entire plate was 1.2%, showing that these assays are robust and have reproducible performance (26). There are MALDI-based clinical assays with regulatory approval considered standard-of-care tests for oncology (27, 28). A MALDI-based assay of note (VeriStrat, Biodesix) uses serum from patients with advanced non–small cell lung carcinoma to determine if patients will have a favorable clinical response to EGFR tyrosine kinase inhibitors (29, 30). This test has been shown to help physicians and impact non–small cell lung carcinoma treatment decisions independent of traditional prognostic factors (31). A similar methodology has been applied to the serum of patients with metastatic melanoma to predict outcomes of Anti-PD-1 therapy (32, 33) and to study the prognostic risk of patients with SARS-CoV-2 (34).

## Clinical Advancements in IMS

Where plate-based assays consider a single mass spectrum for each sample, imaging experiments contain much more data. Translating IMS-based assays into the clinical environment requires stringent controls for sample preparation, data quality, and bioinformatics to withstand regulatory scrutiny. One perceived barrier to translating IMS to the clinical laboratory is the timescale and throughput of image-based assays. Recent technological advances, including sample preparation, instrumentation, and bioinformatics, have dramatically decreased the time needed to perform image-based experiments (35, 36, 37, 38, 39, 40, 41). These advancements aim to create workflows on the same timescale as current routine clinical diagnostics workflows that use histological or antibody stains. For example, samples can be cryosectioned onto slides pretreated with reagents to decrease the time needed for sample preparation (35, 36, 42). Specialized reagents can be used to target specific classes of analytes (43). Analytical instrumentation has advanced to collect data very rapidly, allowing high spatial resolution data to be collected at 20 to 100 pixels/second (38, 44, 45). Ion mobility separations allow for the resolution of some isobaric species, giving further confidence in identifying molecules desorbed directly from tissue sections (41, 44, 46). Postionization lasers (MALDI-2) can increase sensitivity and dynamic range (47, 48).

Although advancements in other analytical methodologies applied to clinical diagnostics have also occurred, there is still a great need for spatially defined molecular approaches in the clinical laboratory. Tissues represent complex environments, with many colocalized cell types and facilitating simultaneous biological processes. The ability to study clinical samples beyond microscopy represents the future of molecular diagnostics.

## Combinations of IMS and microscopy

The correlation of IMS molecular maps with microscopy augments the development of IMS-based clinical assays and is an essential step in translating IMS into a standard pathology tool. An example is using histological stains (H&E, PAS, etc) in combination with IMS data. These technology correlations can be used for qualitative comparisons, such as overlaying images to place molecular information on histological features and for automated cross-modality data mining (49). Most applications of IMS in a clinical assay use some form of staining to guide data interpretation (50, 51) and, in some cases, to guide data acquisition through histology-directed workflows (52, 53, 54).

A consideration in combing microscopy with IMS is that stains applied on the same section after the IMS process can compromise tissue integrity. Label-free microscopy modalities like autofluorescence, where a fluorescence microscopy image of the tissue section is obtained from the tissue's natural fluorescence, provide histological information for data analysis/acquisition (53). Computational methods have recently been demonstrated that use label-free microscopy to “virtually” stain or predict a high-resolution digital pathology image from label-free data (55, 56, 57). Integration of virtual staining approaches with IMS will avoid artifacts caused by staining reagents and allow the selection of targeted regions to image on the same section that will undergo IMS analysis.

The combination of IMS with microscopy has spawned new computational tools and workflows. High accuracy and submicron image alignment between IMS and microscopy has been reported (58). With more combined data, it is equally vital to have seamless multimodal image visualization that enables the best digital pathology features for microscopy with the requirements of IMS’s more complex ion image and spectral visualization (59, 60). With new tools for image alignment and multimodal visualization, the combination of imaging modalities can be expected to become an indispensable tool in the clinic.

## Considerations for the Development of New IMS Assays

As IMS technology advances, the ability to perform spatial assays for specific biological purposes will also proceed accordingly. In addition to routine analysis of lipids, proteins, and peptides, recent advancements have used nontryptic enzymatic digestion or combinations of enzymatic digestions (61). One such example is the use of PNGaseF to enable imaging of N-linked glycans (62, 63), allowing the study of the effects of the role of extracellular matrix in the disease process. N-linked glycans have diagnostic utility and have been shown to differentiate disease, including hepatocellular carcinoma (64) and liver fibrosis (65).

While many IMS studies have discovered biomarkers indicative of specific disease states, there is also the potential to correlate molecular signatures with clinical outcomes for prognostic analysis. A study of oral squamous cell carcinoma determined that the expression of two correlated proteins could be a prognostic factor for patient outcome (66). Another report suggests that the protein thymosin beta-4 can be used as a marker to predict survival in patients with colon cancer (67). Although these studies show that IMS holds the potential to be used in the clinic to diagnose disease and predict outcomes, care is essential in both study design and execution, which affect the quality of the assay and ultimately will determine if it achieves regulatory approval.

## Normalization, Classification, and Scoring

Clinical IMS assays will often depend on complex bioinformatics to find multivariate signatures for the diagnosis and prognosis of diseases. Data generated from samples with known outcomes inform the training of an algorithm to recognize the disease and perhaps stage outcomes from the data alone. Several critical factors must be considered, including data normalization, modeling approach, and scoring criteria. Large clinical data sets typically are not acquired over a short period, necessitating continual instrumental performance evaluation, including signal processing of the mass spectral data such as mass alignment, calibration, and intensity normalization. Measurements across tissue surfaces can experience mass drift, particularly in time-of-flight–based instruments where target orientation can affect the analysis (68). However, it has been shown that chemical background signals in the mass spectra can be used for mass alignment and recalibration (69). Samples of known outcomes are typically used to determine discriminatory peaks, but it is crucial to decide on the criteria used to distinguish those samples. In more traditional approaches, a pathologist’s annotation, clinical follow-up, histological stains, or other ancillary methods are used to provide “ground truth” for the segmentation of tissues (53). In this case, these external data drive the machine learning, and any bias is propagated, but the data are amenable to supervised analysis as all spectra are inherently labeled.

In most clinical studies with IMS, researchers have used well-described machine learning models like linear discriminant analysis or support vector machines directly on peak-picked IMS data (reduction of full-profile mass spectra to only *m/z* bins containing peak centroids). Others have recently described success using 1D convolutional neural networks that forgo any feature filtering before modeling, representing a powerful new means to classify IMS data for diagnostic purposes that removes data filtering biases from peak picking. Recent studies have used machine learning and deep learning to segment IMS data (70, 71, 72). Such unsupervised approaches are data driven and can reveal molecular components that might not be easily distinguished by histology alone but require more mathematical and bioinformatic expertise to implement and interpret correctly (73). Data-driven approaches can segment tissues into similar components, assisting pathologists in annotation (74) and augmenting the understanding of disease microenvironments (70, 75). In another classification approach, Hernandez *et al.* built a classification strategy using known disease markers from a list of 19 proteins commonly analyzed by immunohistochemistry. This targeted IMS study produced higher accuracy than traditional blinded feature-extraction approaches and presented another method for the discriminatory classification of IMS data (76).

In addition to the bioinformatics for creating disease classifiers, care must be taken when defining the parameters of positive and negative results for clinical assays. Because IMS data generate multiple mass spectra from areas of interest and each mass spectrum can be classified, determining how to score samples for clinical assays is only sometimes straightforward. From a statistical perspective, multiple spectra measured from a single tissue section are not independent but can be considered independent at the sample level. A typical scenario can be, “does a single spot classified as a disease amongst a set of negatives indicate a positive diagnosis?” Many studies have generated unique scoring criteria based on data from true positives and true negatives in their validation data (25, 54, 77, 78). These criteria have been unique to each study, and it remains an area of active research that will require input from clinical decision stakeholders and statisticians.

## Data Quality

Batch effects are a problem that can infiltrate clinical omics data collected over time (79). Cross-normalization strategies have been studied for IMS data collected at different centers (69, 80). This approach was reported to reduce batch effects or effects specific to one analytical laboratory. There is robustness built into classifiers when data are generated at multiple sites and using many protocols. Porcari *et al.* used data from two laboratories (Brazil and the United States) to build and test classifiers to diagnose breast cancer. In this study, investigators independently collected a second validation set from a site in Brazil (81). Another multicenter collaboration tested the effects of quantitative IMS from multiple clinical sites and instruments using a mimetic tissue model for quantitative analysis of a small molecule and its first metabolite in the liver (82).

Such studies highlight the need for quality assurance metrics for IMS data. In addition to assay validation at multiple sites, additional metrics need to be in place for quality assurance of MALDI data for clinical assays. The 2019 revision of the College of American Pathologists Chemistry and Toxicology checklist includes new guidelines for IMS data (cap.org). These revised points set standards for instrument calibration, performance evaluation for patient samples, control tissues, analysis areas, data analysis, and data storage (CHM.21405 through CHM.21465). The CAP checklist sets the standard for IMS-based studies in the clinical laboratory and should guide new assay development.

## Regulatory Approval

**Fig. 4:**
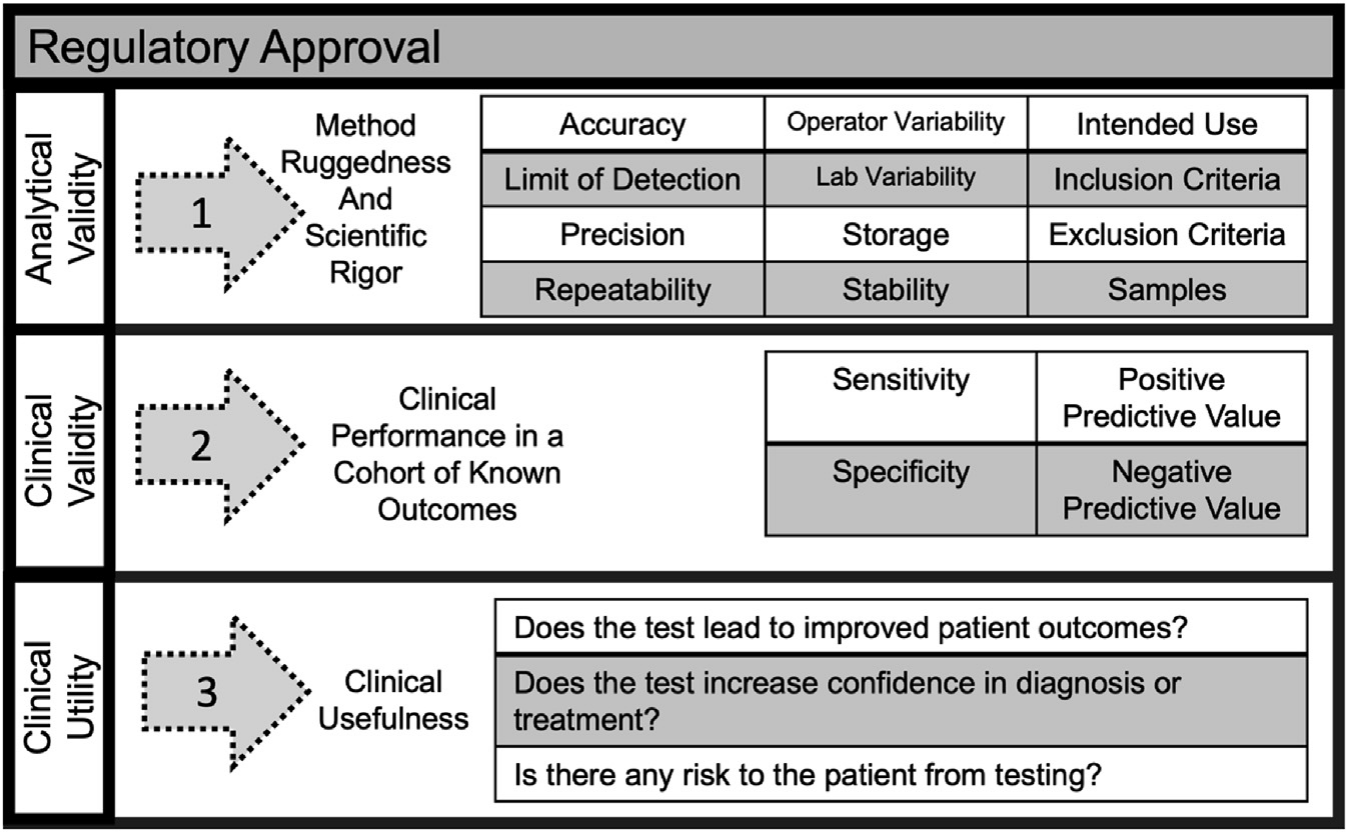
**Overview of the critical elements of regulatory approval for clinical assays.** Regulatory approval requires the assay development process to demonstrate analytical validity, clinical validity, and clinical utility.

The beneficial features of MALDI IMS fit well with the requirements for precision medicine and can be expected to grow alongside other precision medicine assays. This technology requires advanced analytical instrumentation (the mass spectrometer and the sample preparation peripheral instrumentation) and has not yet achieved a fully “turn-key” solution. Thus, at least for the moment, any clinical assay will likely be a “laboratory” test that specialized teams can perform. Figure 4 outlines the different levels of validity a test will need for regulatory approval.

Regulatory approval will rest on a clear description of the workflow and results and a clear definition of assay performance. IMS clinical assays can benefit from the groundwork laid by complex genomic molecular tests that already have regulatory approval processes (83, 84, 85). Genomic assays and other tests are evaluated on three criteria for regulatory approval: analytical validity, clinical validity, and clinical utility (86, 87).

For analytical validity, the technical performance of the test is measured by how well it can routinely perform over time; its analytical sensitivity and specificity, precision, and robustness (over time, operators, sites); and how dependent it is on any particular signal for performance. For IMS, the number of steps involved in sample preparation requires a sample chain of custody, monitoring of instrument performance (both sample preparation and mass spectrometer), and a plan to study a sample or set of samples to benchmark routine performance.

Clinical validity relates to the assay's performance in answering a clinical question, such as a defined presence or absence of clinically defined status (87). For diagnostic applications, this can be the baseline assay performance: does the assay accurately predict the presence of the disease? Essentially, what are its sensitivity and specificity, where sensitivity is the ability to classify disease in known positives and specificity is the ability to differentiate true negatives (88). For prognostic applications, does the assay accurately assess the risk or likelihood of developing severe forms of the disease with poor outcomes? The sensitivity and specificity metrics required to establish clinical validity are very situation dependent and must be evaluated on a case-by-case basis considering the clinical question being addressed. Clinical utility is the synthesis of the previous criteria that assess whether the assay helps clinicians and providers with actionable evidence that changes their course of treatment and whether there is a benefit from using the test. Evaluation of this criterion is critical because it also has implications for the potential success of the assay from a commercial point of view.

Regulatory hurdles for the adoption of IMS assays in the clinical laboratory are still an emerging challenge. The recent introduction of a CAP checklist specific to IMS data is a valuable starting point. It can help emergent studies design rugged enough assays to withstand the regulatory processes, including metrics needed for quality control and assurance.

## Conclusions

IMS is a maturing technology extensively used to study disease. MALDI MS is currently used in the clinical laboratory to classify and identify bacteria and for some other plate-based clinical assays. Expanding this technology to include tissue-based clinical assays to diagnose and prognose disease is the next challenge for the technology. Novel approaches in machine learning have provided robust discriminatory analysis of IMS data and sample scoring. Continued advancements in these areas will increase buy-in from clinical stakeholders and are necessary as IMS is translated into the clinical laboratory.

## Conflict of interest

The authors declare no competing interests.
